# bT_RM_ Control of Murine Cytomegalovirus CNS Reactivation

**DOI:** 10.3390/ijms26115275

**Published:** 2025-05-30

**Authors:** Priyanka Chauhan, Shuxian Hu, Wen S. Sheng, Sujata Prasad, James R. Lokensgard

**Affiliations:** Neurovirology Laboratory, Department of Medicine, University of Minnesota Medical School, Minneapolis, MN 55455, USA; guptap@umn.edu (P.C.); shuxianmn096@gmail.com (S.H.); wenshengsunny@gmail.com (W.S.S.); sujata06@gmail.com (S.P.)

**Keywords:** murine cytomegalovirus (MCMV), tissue-resident memory T-cells (bTRMs), viral reactivation

## Abstract

T lymphocytes infiltrate the CNS in response to murine cytomegalovirus (MCMV) infection and form a pool of long-lived brain tissue-resident memory T-cells (bT_RM_s), which display markers of residency (i.e., CD103, CD69, CD49a). However, the functional role of these bT_RM_s is still unknown. By 30 days postinfection, a latent viral brain infection was established, as indicated by absence of viral transcripts (IE1, E1, and gB) produced during productive infection. Following intracerebroventricular injection of either depleting α-CD8 Ab (clone YTS169.4) or α-CD103-sap (clone IT50) into the brain, 90–95% T-cell depletion was achieved. Using luciferase-expressing mice, we observed recommenced imaging signals indicative of de novo MCMV IE promoter activity in depleted animals. Surprisingly, using an explant assay, we efficiently recovered reactivatable, infectious virus from untreated, latent animals, but not from those depleted of bT_RM_s (viral recovery in explants was reduced from 100% to 50% by day 21). We identified *Lgals3* (galectin 3), *Gpnmb* (glycoprotein nonmetastatic melanoma protein B) and *Hmox1* (heme oxygenase 1) as genes that were most upregulated in bT_RM_-depleted groups. When bT_RM_s were depleted, there was transient expression of viral IE genes which resulted in antiviral microglia with a phagocytic, disease-associated (DAM) or neurodegenerative (MGnD) phenotype. These data provide new insights into the role of bT_RM_s in controlling both CNS reactivation and driving microglial phenotypes.

## 1. Introduction

Previous work from our laboratory has demonstrated that neural stem cells (NSCs) in the sub-ventricular zone (SVZ) and hippocampus are primary targets for murine cytomegalovirus (MCMV, a β-herpesvirus) during acute viral brain infection [1,2,3]. However, following the acute phase, a subset of these infected NSCs survive and subsequently develop into mature neurons which harbor latent MCMV genomes. Subsequent studies using explant cultures of brain tissue have shown that reactivated virus can then be grown out of this neuronal reservoir months after primary infection [4]. The resistance of neurons to cytotoxic T-cell (CTL) activity makes them a hospitable host cell for harboring long-term, latent infections. But without reactivation, latent infections likely would not cause problems. However, repeated cycles of reactivation from latent central nervous system (CNS) reservoirs, along with corresponding de novo viral protein synthesis, is a likely source of foreign antigen within the brain, which may drive chronic neuroinflammation.

Infections of the brain elicit neuroimmune responses that are necessary to limit viral spread, and data from our laboratory have shown that CD8^+^ T-cells infiltrate the brain in response to primary viral infection and, subsequently, form a pool of long-lived, brain-resident memory T-cells (bT_RMs_) which display markers of tissue residency (e.g., CD103, CD69, CD49a) [5,6]. Although the functional role of these brain-resident T-cells is unknown, an appreciation of the deleterious consequences of viral reactivation-driven inflammation on chronic neurodegenerative disease is beginning to emerge.

Because *bona fide* bT_RMs_ do not express tissue residency markers within the CNS until after acute, productive infection has been resolved i.e., 14–21 days post infection (d p.i.), these findings suggest that bT_RMs_ function during the later stages of brain infection [6]. A previous study by Brizic et al., showed that adoptive transfer of virus-specific T-cells protected newborn mice against MCMV infection, and that depletion of bT_RMs_ resulted in virus reactivation and enhanced neuroinflammation [7]. So, the current study was undertaken to further understand the role of bT_RMs_ in controlling reactivation of MCMV in our brain infection model. To this end, we employed FVB transgenic mice which express luciferase under the RosA26 promoter as well as explant assay, the gold standard to assess production of infectious, reactivated virus.

Microglial cell phenotypes are constantly being shaped by their particular brain microenvironment, such as those induced by bT_RMs_ or viral infection. Although over a dozen alternative microglial activation phenotypes have been described, a prototypic microglial alternate activation phenotypic signature (i.e., disease-associated microglia [DAM]) has been identified in a variety of common neurodegenerative diseases [8]. The DAM signature is elicited by the triggering receptor on the myeloid cells 2 (TREM2) pathway [9], which activates neurodegenerative markers (e.g., GPNMB, LGALS3) and also promotes phagocytosis [10], while at the same time suppressing markers of the homeostatic phenotype (e.g., the purinergic receptor P2RY12). In response to specific brain microenvironments, microglia first mature into stage 1 DAM and upregulate TREM2. Subsequent full differentiation into stage 2 DAM is TREM2-dependent. These DAM cells are generally believed to be protective because they are highly phagocytic. So, we went on to investigate glial cell gene expression during bT_RM_ depletion and their involvement in inducing phagocytic, antiviral microglia.

## 2. Results

### 2.1. Viral Expression and Establishment of Latency Following MCMV Infection

**Figure 1:**
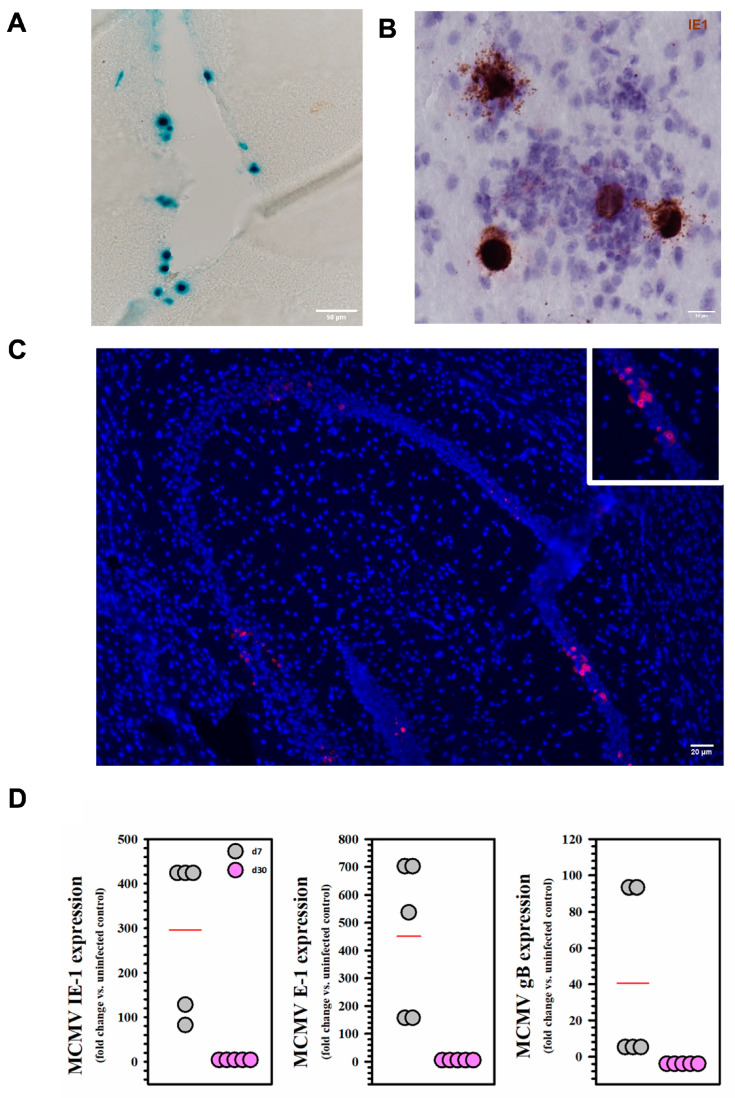
Viral expression and establishment of latency following MCMV infection. Mice were infected intracerebroventricularly with 1 × 10^5^ TCID_50_ units (in 10 μL) of MCMV. At 5 d p.i., brain tissues from infected animals were harvested. (**A**) X-gal staining of β-gal-expressing virus (MCMV RM461) in the infected brain. (**B**) Staining for IE1 viral transcripts using RNAscope (brown). (**C**) Positive RNAscope staining for viral nucleic acid at D30. (**D**) Expression of IE1, E1, and gB transcripts was assessed during both acute (d7) and latent infection (d30) using real-time RT-PCR. Each dot represents RNA extracted from one animal (5 mice/timepoint).

BALB/c mice were infected with MCMV via the intracerebroventricular (icv) route and analyzed for the presence of viral promoter activity, DNA, or RNA at 7 d and 30 d post infection (p.i.). We first detected X-gal staining indicative of β-galactosidase expressing virus in the infected brain at 5 d p.i. (Figure 1A). This observation was confirmed using RNAscope at 5 d p.i., where we detected RNA specific for MCMV IE-1 (Figure 1B). Using similar techniques, we also observed MCMV nucleic acid at 30 d p.i. (Figure 1C). Furthermore, we carried out qRT-PCR experiments using brain tissue harvested at both 7 d (acute phase) and 30 d p.i. (latent phase). Expression of all classes of viral genes (IE-1, E-1, and gB) was observed at 7 d, but not at 30 d p.i. (Figure 1D).

### 2.2. Co-Localization of CD8^+^ T-Cells and MCMV E1-Retaining Cells

**Figure 2:**
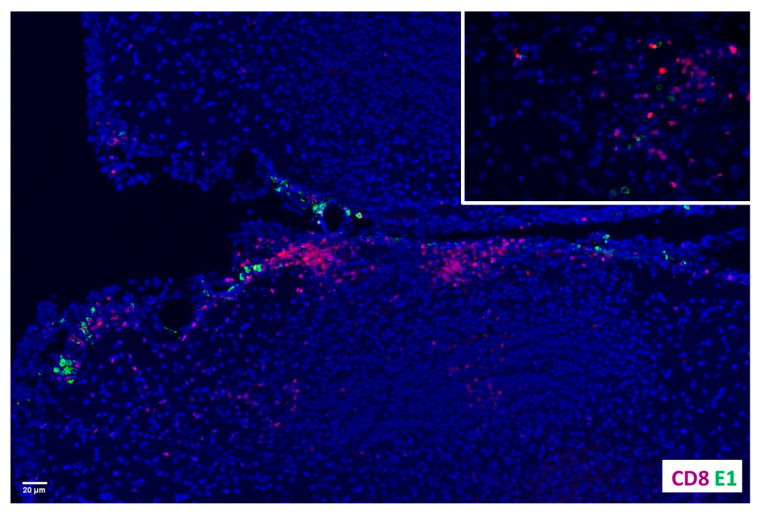
Co-localization of CD8^+^ T-cells and MCMV E1-retaining cells. Brain sections of mice infected with MCMV for 30 d were double-stained for MCMV E1 and CD8 and viewed using fluorescent microscopy. Red indicates α-CD8 staining while green displays α-E1.

We went on to assess the localization of CD8^+^ T-cells near latently infected cells. Previous studies using neonatal mice reported that the MCMV early gene E1 (m112–113) product has a tendency to be retained in at least some neurons following MCMV-infection [11], and its presence can be detected for a prolonged time [12]. We previously obtained this monoclonal α-E1 antibody (Ab), (kindly provided by Dr. I. Kosugi, Hamamatsu University, Japan), and used it to successfully stain MCMV infected cells at extended times (i.e., 30 d) following resolution of acute infection [4]. Here, this Ab was used to co-localize CD8^+^ bT_RMs_ with MCMV E1-retaining cells (Figure 2). These findings suggest that bT_RM_ neuronal interactions may affect viral reactivation and recurrence.

### 2.3. Depletion of CD8^+^ and CD8^+^CD103^+^ T-Cells from the Brains of MCMV-Infected Animals

**Figure 3:**
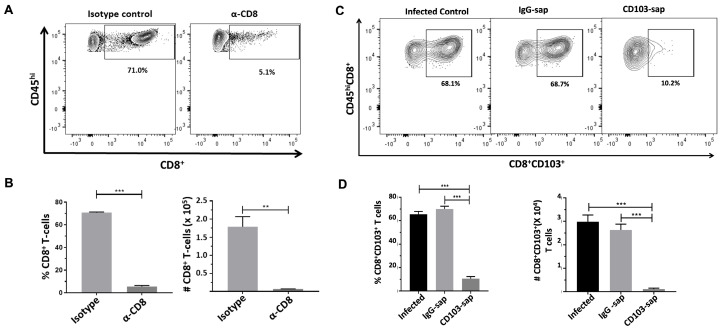
Depletion of CD8^+^ and CD8^+^CD103^+^ T-cells from the brains of MCMV-infected animals. Mice were infected with 1 × 10^5^ TCID_50_ units (in 10 μL) via icv injection. At 30 d p.i., mice were injected with either α-CD8 depleting antibody [YTS-169.4 (65 µg)] or its isotype control [LTF-2 (65 µg)], α-CD103-sap [2 µg, IT-50], or its isotype control, IgG-sap [2 µg, IT-17]. At 3 d post-injection, mice were euthanized and brain tissues were harvested to isolate brain mononuclear cells (BMNCs), which were labeled with Abs specific for anti-CD45-PE-Cy7, anti-CD8-BV510, and anti-CD103-FITC for analysis by flow cytometry. (**A**) Representative contour plots show the percentages of CD8^+^ T lymphocytes within depleted (α-CD8 treated) and non-depleted groups (isotype control). (**B**) Frequency and the number of CD8^+^ T-cells belonging to the indicated groups. (**C**) Representative contour plots show the percentages of CD8^+^CD103^+^ T lymphocytes within infected control, IgG-sap treated, and α-CD103-sap treated animals. (**D**) Frequency and number of CD8^+^CD103^+^ T-cells belonging to the indicated group. Pooled data present absolute numbers (mean ± SE) of CD8^+^ and CD8^+^CD103^+^ T-cells from two independent experiments using 4–6 animals per group. ** *p* < 0.01, *** *p* < 0.001.

To assess the role of bT_RMs_ (CD8+CD103+) in controlling latent viral reactivation, we went on to deplete these cells from the brains of latently infected mice. Mice latently infected with MCMV were injected with either α-CD8 depleting antibody or α-CD103-sap. At 3 d post-injection, mice were euthanized and brain tissues were harvested to isolate brain mononuclear cells (BMNCs) which were then analyzed for the presence of CD8 + T-cells or CD8+CD103+ T-cells by flow cytometry. We observed only 5.1% of cells to be CD8+ following depletion, as compared to 71.0% in the non-depleted group (Figure 3A,B). Similarly, we observed 10.2% of CD8+CD103+ T-cells in animals injected with α-CD103-sap as compared to the control (non-depleted and IgG-sap groups) animals (Figure 3C,D).

### 2.4. Establishment of Latent cre-MCMV Infection in FVB Transgenic Mice Using Bioluminescent Imaging

**Figure 4:**
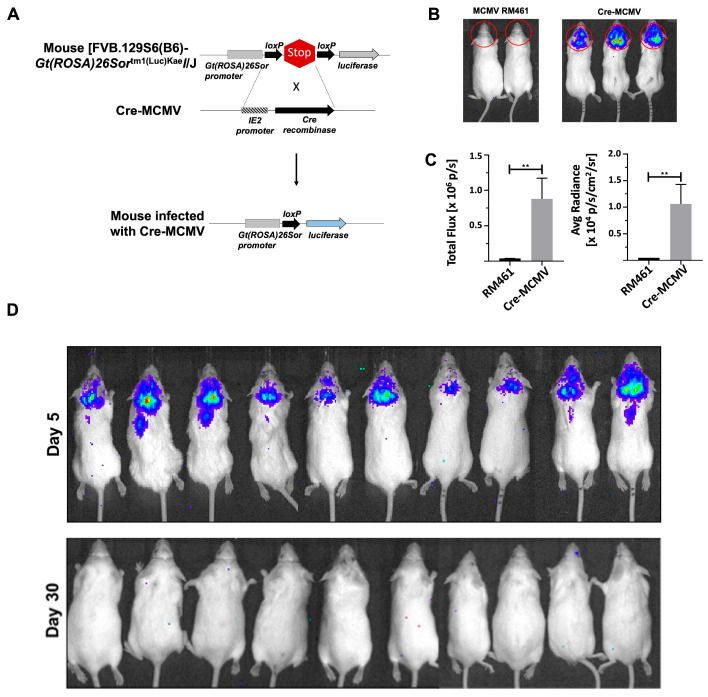
Bioluminescent imaging of *cre*-MCMV-infected FVB transgenic mice and establishment of latent *cre*-MCMV infection. (**A**) Schematic outline. FVB transgenic mice that express luciferase under the ROSA26 promoter, but have a stop signal flanked by LoxP sites, were infected with *cre*-MCMV, a virus which expresses Cre recombinase under control of the IE2 promoter (i.e., during productive infection). (**B**) Infected animals were imaged at day 5 p.i. using an IVIS 100 after injecting 150 µg of D-luciferin (i.p.). Animals were imaged 5 min after D-luciferin administration and data were acquired using a 5-min exposure window. Bioluminescence imaging was conducted using *cre*-MCMV-infected as well as β-gal expressing MCMV-infected FVB mice. Red circle is the area from which the signal is quantified. (**C**) Data were analyzed using Living Image software. (https://www.revvity.com/category/in-vivo-imaging-software, (accessed on 25 May 2025) Revvity, San Diego, CA, USA). (**D**) Luciferase-expressing FVB transgenic mice were infected with *cre*-MCMV and imaged at days 5 and 30 p.i. The figure shows pooled data from two independent experiments using five animals each. ** *p* < 0.01.

To longitudinally examine CNS viral reactivation, we established a latent MCMV infection in FVB transgenic mice containing a luciferase reporter gene but flanked by stop signals (Figure 4A). For these experiments, we used a Cre recombinase-expressing recombinant MCMV virus, where *cre* is inserted into the MCMV IE2 locus to infect the FVB transgenic mice (Figure 4A). Mice infected with *cre*-MCMV exhibited a strong signal both quantitatively and qualitatively on imaging, as observed in Figure 4B,C, while mice infected with MCMV RM461 failed to exhibit any signal. Further, we expanded the experiment using 10 mice and found infection at day 5 p.i. in all the mice as observed by a strong signal. Importantly, upon imaging these mice at day 30 p.i., we did not observe any signal (Figure 4D); which indicated the establishment of latent infection.

### 2.5. Role of CD8 or CD103 in Controlling Viral Reactivation Within the Brain

**Figure 5:**
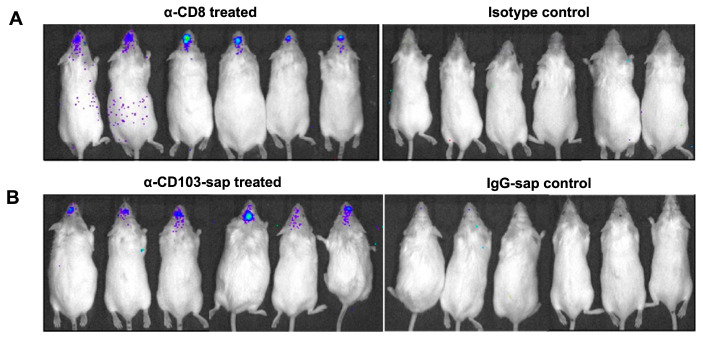
Role of CD8 or CD103 in controlling viral recrudescence within the brain. (**A**) Latently-infected FVB transgenic mice were injected with either α-CD8 or its isotype control 30 d following infection with *cre*-MCMV. Animals were subsequently imaged longitudinally at various times post-antibody injection; day 10 post-injection is shown. (**B**) After 30 d of *cre*-MCMV infection, FVB mice were injected with either α-CD103-sap or IgG-sap control. Animals were imaged at day 10 post-T-cell depletion. Pooled data from two independent experiments using three animals each are shown.

To further assess the role of CD8+ and CD103+ T-cells in controlling viral reactivation, we then established latency in FVB transgenic mice (infected with *cre*-MCMV), as demonstrated in Figure 4. These latently infected mice were then depleted of CD8 and CD103 by injecting α-CD8 and α-CD103-sap and monitored longitudinally for luciferase expression by bioluminescent imaging. We observed luciferase expression in the latently infected transgenic mice following 10 d of CD8+ T-cells, as well as following CD103+ T-cell depletion (Figure 5A,B).

### 2.6. Recovery of Reactivated Infectious Virus from the Brains of Latently-Infected Animals Depleted of bT_RM_

**Figure 6:**
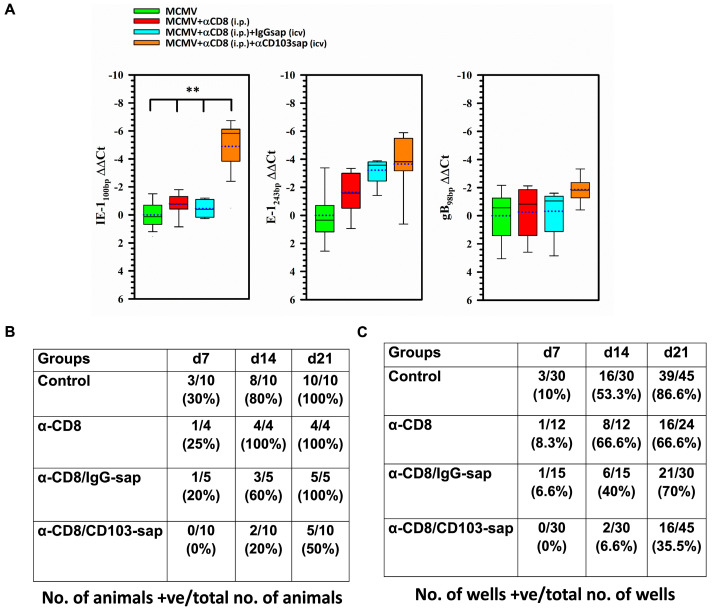
Viral gene expression and recovery of reactivated, infectious virus from the brains of latently-infected animals following bT_RM_ depletion. Periventricular brain tissue obtained from latently-infected animals (30 d p.i.) was cut into 1 mm pieces and placed onto primary murine glial cell cultures. (**A**) At 3 d post-explant, these cultures were processed for real-time PCR for MCMV IE1, E1, and gB transcripts. (**B**,**C**) At 7, 14, and 21 d post-explant, these cultures were monitored for reactivated virus by cytopathic effect and β-gal expression. ** *p* < 0.01.

To further analyze the role of bT_RM_ in controlling viral recrudescence, we employed a classic explant assay. For this, we infected BALB/c animals and allowed the virus to go latent. A group of these animals was then treated with α-CD8 alone or injected with α-CD8 intraperitoneally followed by icv injection of either IgG-sap or CD103-sap. Three days post-depletion, the brains were collected and chopped into fine pieces. Some pieces of brain were used to study the expression of viral genes using qRT-PCR while the rest were co-cultured with mixed glial cells and analyzed by X-gal staining at day 7, 14, and 21 post-explant. In these experiments, we observed significant MCMV IE expression in animals depleted of bT_RMs_ (Figure 6A). However, we did not observe reactivated virus by explant culture at 7 d post-bT_RM_ depletion (0% as compared to 20–30% in control groups). Similar results were obtained at later time points. Surprisingly, although a higher level of IE gene expression was observed in the bT_RM_-depleted animals (Figure 6A), the recovery of reactivated, infectious virus was paradoxically less in animals following bT_RM_ depletion at 14 d (i.e., in 20% of animals as compared to 60–100% in the three control groups), (Figure 6B,C). At 21 d post-explant, viral reactivation was observed in 50% of bT_RM_-depleted animals compared to 100% of the animals in each of the control groups.

### 2.7. Profiling Brain Microenvironments

**Figure 7:**
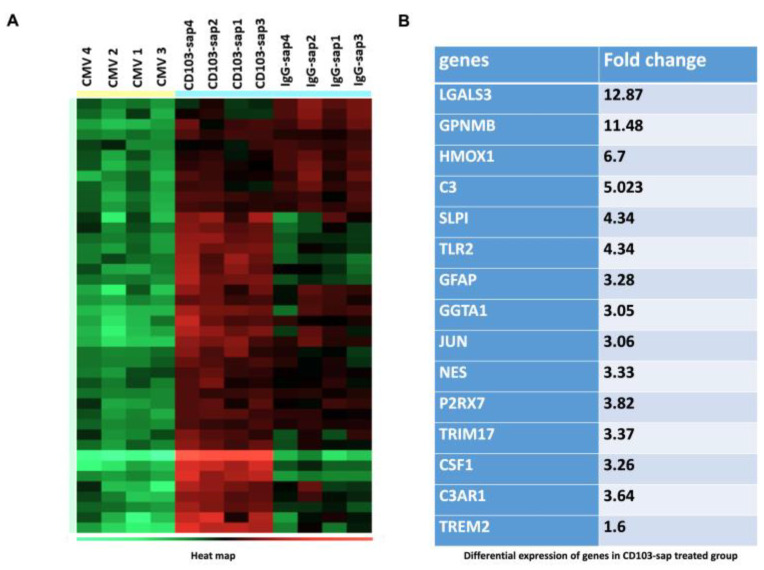
Profiling brain microenvironments. RNA was extracted using Qiagen RNeasy Lipid Tissue extraction kit from the brains of mice latently infected (>30 d p.i.) with MCMV either treated with CD103-depleting Ab (CD103-sap) or IgG control (IgG-sap) or left untreated (CMV) 3 d post-depletion. RNA was analyzed using the NanoString nCounter Glial Cell panel and Rosalind Bioinformatics Online Platform Software (https://www.rosalind.bio/). (**A**) Heat map depicting glial cell profiling using the nCounter panel. (**B**) List of the most differentially expressed genes in CD103-depleted group.

To investigate the effects of bT_RM_ depletion on brain-resident cells, we carried out nCounter Glial Cell Profiling using Nanostring technology, and the resulting data were analyzed using ROSALIND. We compared RNA extracted from the brains of latently infected animals to those of latently infected animals following in vivo depletion of CD103^+^ bT_RMs_. Taken together, we observed a total of 84 genes that were differentially expressed between the depleted and undepleted groups, 30 of which belong to the DAM, or neurodegenerative microglia (MGnD), core theme annotation, including *Lgals3*, *Gpnmb*, *Csf*-1 (colony stimulating factor 1), and *Trem2* (triggering receptor on myeloid cells 2) in the bT_RM_-depleted groups (Figure 7A,B).

## 3. Discussion

We first demonstrate that acute MCMV brain infection is followed by establishment of latency within the CNS. Herpesvirus gene expression can be classified as immediate early (alpha), early (beta), or late (gamma) according to their temporal order of expression. Upon initial infection, we saw expression of all classes of viral genes (i.e., IE1, E1, and gB) at 7 d (acute phase), but not 30 d p.i. When RNA extracts of brain homogenates were examined at this later time point, it was apparent that there was a true latent infection within the brain, as opposed to a chronic productive infection, because transcription of none of these gene classes was detected. Despite the absence of detectable viral IE1, E1, or gB transcripts using real-time RT-PCR, the latent viral nucleic acid was still detectable within neurons, as indicated by positive RNAscope staining. Using this same animal model, we have previously shown the presence of CD8+ T-cells within the brain, and further determined that they form a pool of brain-resident memory T-cells which persist for a lifetime [5,6].

Previous studies, using neonatal mice, reported that the MCMV early gene E1 (m112–113) product has a tendency to be retained in at least some neurons following MCMV-infection [11], and its presence can be detected for a prolonged time [12]. We previously obtained this monoclonal α-E1 Ab (kindly provided by Dr. I. Kosugi, Hamamatsu University, Japan), and used it to successfully stain MCMV infected cells at extended times following resolution of acute infection [4]. In this study, we show that CD8+ T-cells residing within the brain at latent time points co-exist with MCMV E1-retaining cells using fluorescent microscopy. These findings suggest that bT_RM_—neuronal interactions may affect viral control. To test this hypothesis, we went on to deplete CD8+ and CD103+ T-cells in the brain through the injection of depleting anti-CD8 and anti-CD103-saporin Abs, respectively. Depletion was achieved via ICV injection of either α-CD8 Ab (clone YTS169.4) or α-CD103-sap (clone IT50), which is an Ab linked to the saporin toxin (Advanced Targeting Systems, Carlsbad, CA, USA), into the latently-infected brain. Both of these methods achieved 90–95% T-cell depletion. This depletion was then used to analyze the role of these cells in viral reactivation, through imaging experiments and explant assays.

To longitudinally examine real-time viral reactivation in the brains of infected animals, groups of LucRep reporter gene-containing transgenic mice were infected with the *cre*-MCMV virus (Figure 4). A similar approach has previously been shown to be useful in studies of herpes simplex virus-1 [13] and murine gammaherpesvirus 68 (MHV-68), [14]. Using the Cre recombinase expressing virus in imaging experiments, we successfully demonstrated acute infection in transgenic FVB mice where the STOP signal was excised by virus-expressed Cre recombinase. Importantly, the infection was found to go latent as indicated by the absence of imaging signal at day 30. These data indicate that luciferase-expressing cells during acute infection were cleared following acute infection.

Latent Cre-expressing MCMV was found to be reactivated following bT_RM_ depletion. When the signal from the acute phase was no longer detectable via bioluminescent imaging, we longitudinally imaged the animals every other day for two weeks to assess viral reactivation and determine optimal time points for further studies. It should be noted that the imaging signal observed could be due to the presence of only IE promoter activity without further viral gene expression (i.e., indicative of an abortive full productive infection). Evidence exists to support the idea that limited expression of MCMV T-cell epitope-encoding genes during reactivation events can lead to recognition of the reactivating target cells by T lymphocytes even before the productive cycle is completed and infectious virions are produced [15,16,17]. In two of the anti-CD8-treated animals, it appears that the luciferase activity may have spread beyond the CNS (Figure 5A). This signal was initially dismissed as background, but it is possible that reactivated virus spread from the CNS following depletion of the CD8+ T-cells.

We examined this possibility using an explant assay. We found that explant reactivation of brain tissue obtained from animals following bT_RM_ depletion resulted in de novo expression of IE1 transcripts, as determined using real-time RT-PCR. The detection of IE gene expression, but not the later classes of viral genes, in the explant assay following CD103+ T-cell depletion could suggest that viral reactivation was blocked or aborted prior to the full production of infectious particles (Figure 6). While imaging and transcriptional analysis provide valuable information regarding viral reactivation, actually isolating infectious virus from latent brain tissue using explant cultures is the “gold standard” for assessing reactivation of infectious virus. Through comparison of viral explant reactivation in groups of animals with and without T-cell depletion, the role of bT_RMs_ in confining productive reactivation following brain explant was investigated. Latent virus can be grown out of the brains of undepleted control animals (100% by 21 d, Figure 6C) using explant culture alone. Paradoxically, in animals depleted of bT_RMs_, we found viral reactivation kinetics to be delayed when compared to undepleted controls. We observed no CPE indicative of productive virus in any of the 10 animals examined at 7 d post-explant. However, given sufficient time (i.e., 21 d) following explant, virus was recovered from 50% of the bT_RM_-depleted animals (vs. 100% in each control group).

It is possible that neurons expressing IE proteins (i.e., those produced first upon viral reactivation) are quelled by viral-specific brain-resident memory CD8+ T cells and thereby prevent subsequent gene expression and viral reactivation. Unexpectedly, it appears that the signal observed during imaging experiments did not translate into a productive infection as indicated by our explant study. So, we hypothesize that when we depleted bT_RMs_, there was transient viral expression of at least IE genes, which was sufficient to activate surveying microglia and induce phagocytic glial cell phenotypes resulting in a tissue-wide anti-viral state. Although over a dozen alternative microglial activation phenotypes have been described, disease-associated microglia (DAM), which were first identified in Alzheimer’s disease, are observed in neurodegenerative models [8]. But, in addition to slowly progressive neurodegenerative diseases, recent studies have shown that cells of the DAM phenotype also develop following a number of viral brain infections (e.g., ZIKV, WNV, HIV-1, and HSV-1) [18,19,20]. Microglia of the DAM phenotype are generally believed to be protective because they are highly phagocytic, but it is likely that overzealous or aberrant DAM-mediated synaptic elimination is harmful for cognitive function. One caveat is that the NanoString data obtained may not be exclusive to microglial cell populations since RNA was extracted from whole brain. These data were generated using nCounter Glial Cell Profiling, which also includes prototypical astrocyte genes.

Although still controversial, neurodegenerative diseases following the reactivation of latent herpesvirus infections from neuronal reservoirs, and induced by the ensuing neuroinflammatory responses, have been associated with a number of human herpesviruses, including cytomegalovirus (CMV) and human herpesvirus (HHV)-6, in addition to herpes simplex virus (HSV)-1 [21,22,23,24,25,26,27,28,29,30]. Although extrapolation regarding the role of TRMs in human neurodegenerative diseases in the absence of direct evidence needs to be tempered, upon reactivation of quiescent neurotropic viruses, bT_RMs_ likely respond to de novo-produced viral Ag through rapid release of interferon (IFN)-γ [31]. Through this mechanism, a small number of adaptive bT_RMs_ may amplify responses to viral proteins produced during reactivation, leading to an organ-wide innate protective state [32,33,34]. In the absence of bT_RMs_, the replication cycle is allowed to proceed and brain-resident glia likely respond by phagocytosing the increased levels of foreign viral protein. Over time, this protective phagocytic immune activation may have cumulative neurotoxic and neurocognitive consequences [35].

## 4. Materials and Methods

### 4.1. Ethical Statement

This study was carried out strictly in accordance with recommendations in the Guide for the Care and Use of Laboratory Animals of the National Institutes of Health. The protocol was approved by the Institutional Animal Care and Use Committee (Protocol Number: 2211-40536A, approval date: 1 October 2023) of the University of Minnesota. All animals were routinely cared for according to the guidelines of Research Animal Resources (RAR), University of Minnesota. All surgery was performed under Ketamine/Xylazine anesthesia and Bupivacaine administration and all efforts were made to ameliorate animal suffering. Animals were sacrificed after isoflurane inhalation, whenever required.

### 4.2. Experimental Animals

BALB/c (8 weeks old) mice were infected with β-gal-expressing MCMV, while FVB transgenic mice (LucRep) were infected with *cre*-expressing MCMV. Pathogen-free BALB/c (stock #028), and FBV transgenic (FVB.129S6(B6)-Gt (ROSA)26Sor^tm1(Luc)Kael^/J, stock #005125) mice were purchased from Charles River Laboratories (Wilmington, MA, USA) and The Jackson Laboratories (Bar Harbor, ME, USA), respectively. Animals (4–5 per cage) were housed on a rack (7 columns × 8 rows) in the animal room under a 12-h light-dark cycle (6 a.m.–6 p.m. light) with free access to standard chow and water. Each rack had 2 cages of sentinel mice for periodic pathogen screening. Animals were checked daily, and cages were changed every week by RAR staff. Mice were acclimated for a minimum of one week prior to research use and weighed approximately 21 g at 8 weeks old.

### 4.3. Virus and Growth Conditions

RM461, a recombinant MCMV expressing *E. coli* β-galactosidase under the control of the human ie1/ie2 promoter/enhancer, was kindly provided by Edward S. Mocarski. *cre*-MCMV, a Cre-recombinase expressing recombinant virus in which *cre* is inserted into the MCMV IE2 locus, was a generous gift from Luka Cicin-sain (Helmholtz Center for Infection Research, Braunschweig, Germany) [36]. Viral stocks were passaged in salivary glands of weanling Balb/c mice to retain their virulence. Virus isolated from the salivary glands was then passaged twice on NIH 3T3 fibroblasts to minimize any carry-over of salivary gland tissue. Infected 3T3 cultures were harvested at 80% to 100% cytopathic effect and subjected to three freeze–thaw cycles. Cellular debris was removed by centrifugation (1000× *g*) at 4 °C, and the virus was pelleted through a 35% sucrose cushion (in Tris-buffered saline [50 mM Tris–HCl, 150 mM NaCl, pH 7.4]) at 23,000× *g* for 2 h at 4 °C. The pellet was suspended in Tris buffered saline containing 10% heat-inactivated fetal bovine serum (FBS). Viral stock titers were determined on 3T3 cells as 50% tissue culture infective doses (TCID_50_) per milliliter. This sucrose gradient-purified RM461 and *cre*-MCMV were used for ICV infections of mice.

### 4.4. Intracerebroventricular (icv) Infection of Mice

Infection of BALB/c mice and FVB transgenic mice with MCMV RM461 and *cre*-MCMV, respectively, was performed as previously described. Briefly, female mice (8 weeks old) were anesthetized using a combination of Ketamine (100 mg/kg body weight; Akorn Inc., Lake Forest, IL, USA) and Xylazine (10 mg/kg body weight; Bimeda Inc., Le Sueur, MN, USA) and immobilized on a small animal stereotactic instrument equipped with a Cunningham mouse adapter (Stoelting Co., Wood Dale, IL, USA). Skin was sterilized using a Betadine solution (Stamford, CT, USA) and subcutaneous injection of the analgesic bupivacaine (Hospira Inc., Lake Forest, IL, USA); [1–2 mg/kg (0.4–0.8 mL/kg of a 0.25% solution)] was administered in the head area prior to incision to ameliorate pain. The skin and underlying connective tissue were reflected to expose reference sutures (sagittal and coronal) on the skull. The sagittal plane was attuned so that bregma and lambda were positioned at the same coordinates on the vertical plane. A burr hole was drilled at pre-determined co-ordinates (AP = 0.9 mm, ML = 0.5 mm from bregma, and DV = 3.0 from skull surface) to access the right ventricle. Animals were injected with virulent, salivary gland-passaged MCMV RM461 (1.0 × 10^5^ TCID_50_ units in 10 μL) or *cre*-MCMV (2.5 × 10^4^ TCID_50_ units in 10 μL) into the ventricles using a 10 µL Hamilton syringe fitted to a 27 G needle over a period of 5 min. The opening in the skull was sealed with sterile bone wax (Guaynabo, Puerto Rico) and the skin was closed using 4–0 silk sutures with a FS-2 needle (Ethicon, Somerville, NJ, USA).

### 4.5. Isolation of Brain Leukocytes and Flow Cytometric Analysis

Brain mononuclear cells were isolated from MCMV-infected BALB/c mice using a previously described procedure with minor modifications [37,38,39]. In brief, whole brain tissues were harvested, (*n* = 4–6 animals/group/experiment), and minced finely using a scalpel in RPMI 1640 (2 g/L D-glucose and 10 mM HEPES) and digested in 0.0625% trypsin (in Ca/Mg-free HBSS) at room temperature for 20 min. Single cell preparations of infected brains were suspended in 30% Percoll and banded on a 70% Percoll cushion at 900× *g* for 10 min at 15 °C. Brain leukocytes obtained from the 30–70% Percoll interface were collected. Following preparation of single cell suspensions, cells were treated with Fc block (anti-CD32/CD16 in the form of 2.4G2 hybridoma culture supernatant with 2% normal rat and 2% normal mouse serum) to inhibit nonspecific Ab binding. Cells were then counted using the trypan blue dye exclusion method, and 1 × 10^6^ cells were subsequently stained with anti-mouse immune cell surface markers for 15 min at 4 °C (anti-CD45-PE-Cy7, anti-CD8-FITC, anti-CD103-PE (eBioscience, San Diego, CA, USA), anti-MHCII-BV510 (BioLegend, San Diego, CA, USA). A total of 10^5^ cells were acquired per sample by using a FACS Fortessa flow-cytometer by employing FACS DIVA software v8.0 (BD Biosciences, San Jose, CA, USA). Data were analyzed using FlowJo software v10.10.0 (Ashland, OR, USA).

### 4.6. Bioluminescence Imaging

Expression of firefly luciferase was monitored by imaging live animals using an IVIS100 (Xenogen; now PerkinElmer, Waltham, MA, USA) equipped with a charge-coupled camera device. Briefly, 150 µg of D-luciferin (Gold Biotechnology, St. Louis, MO, USA) was injected in mice intraperitoneally 5 min before imaging. Data were acquired using a 5-min exposure window.

### 4.7. Semi-Quantitative RT-PCR

Total RNA from infected brain tissue was extracted using a RNeasy Lipid Tissue Mini Kit (Qiagen, Valencia, CA, USA) according to the manufacturer’s recommendations. The cDNA was synthesized from DNase-treated total RNA (1 μg) using Superscript III reverse transcriptase, RNase inhibitors (Invitrogen, Carlsbad, CA), and oligo d(T)_12–18_ primers (Gene Link, Hawthorne, NY). PCR was performed with the SYBR Advantage qPCR premix (Takara Bio USA, Mountain View, CA). The qPCR conditions were as follows: 1 denaturation cycle at 95 °C for 10 s; 40 amplification cycles of 95 °C for 10 s, 60 °C annealing for 10 s, and elongation at 72 °C for 10 s, followed by 1 dissociation cycle (Bio-Rad CFX96 qPCR System, Hercules, CA, USA). The relative expression levels were quantified using the 2^−∆∆Ct^ method [40] and were normalized to the housekeeping gene hypoxanthine phosphoribosyl transferase (HPRT). The primer sequences were 5′-ATCTGAAACAGCCGTATATCATCTTG-3′ sense and 5′-TCAGCCATCAACTCTGCTACCAAC-3′ antisense for IE-1; 5′-GTAAGCACGCAAGCAAGCACT-3′ sense and 5′-CAGAGGGGGACCAGGGATAATA-3′ antisense for E-1 and 5′-GCTGTTTTAACGCGCGGAGTATCA-3′ sense and 5′-TGACGATTCGGGTAAGGCGTGGACTA-3′ antisense for gB.

### 4.8. Immunohistochemistry

Brains were harvested from MCMV-infected animals that were perfused with phosphate-buffered saline (PBS), 2% sodium nitrate to remove contaminating blood cells, and prefixed with 4% paraformaldehyde. Murine brains were subsequently submerged in 4% paraformaldehyde for 24 h and transferred to 25% sucrose solution for 2 d prior to sectioning. After blocking (10% normal goat serum and 0.3% Triton X-100 in PBS) for 1 h at room temperature (RT), brain sections (30 µm) were incubated overnight at 4 °C with the following primary antibodies: monoclonal anti-E1 antibody (MAb kindly provided by Dr. I Kosugi, Hamamatsu University, Hamamatsu, Japan) and monoclonal anti-CD8 antibody. Brain sections were washed three times with PBS and then incubated with fluorescein (FITC)–conjugated anti rat antibody (1:200; Vector Laboratories, Burlingame, CA), or Cy3-conjugated streptavidin (1:400; Jackson ImmunoResearch Laboratories) for 1 h at RT.

### 4.9. RNAscope™ ISH

Commercially available MCMV target probes and reagents were purchased from Advanced Cell Diagnostics (ACD Bio, now Biotechne). Parafilm embedded sections were heated, dewaxed, and dehydrated. RNAscope Pretreat 1 reagent (endogenous peroxidase block) was used to incubate slides for 10 min at room temperature, followed by boiling slides in RNAscope Pretreat 2 buffer (citrate buffer [10 nmol/L, pH 6]) for 30 min; they were then washed, dehydrated, and air dried. Slides were incubated in diluted RNAscope pretreat 3 reagent (protease digestion solution; 2.5 ug/mL) for 20 to 25 min at 40 °C in a HybEZ hybridization oven. After rinsing with water, the slides were incubated with pre-warmed target probes (20 nmol/L of each oligo probe) in hybridization buffer A (6X SSC [1XSSC is 0.15 mol/L NaCl, 0.015 mol/L Na-citrate], 25% formamide, 0.2% lithium dodecyl sulfate, blocking reagents) for 2 h at 40 °C. After washing with buffer (0.1X or 0.05X SSC, 0.03% lithium dodecyl sulfate), slides were incubated with amplification reagents, as described in the RNAscope 2.0 HD detection protocol. Since Amplification 6 contained alkaline phosphatase, chromogenic detection was performed using FastRed as a substrate to generate a red signal and DAB (ImmPACT™ DAB, Vector Laboratories) to generate a brown signal. Slides were counterstained with haematoxylin, mounted in Permount, scanned, and photographed under the microscope.

### 4.10. Explant Assay

Brain tissues were isolated from latently infected mice with or without bT_RM_ depletion. One mm coronal sections were cut using a precision brain matrix (Braintree Scientific, Braintree, MA, USA). Approximately 25 pieces were collected from each brain that represented tissue adjoining lateral and central ventricles. Three to four pieces were then inoculated onto previously prepared primary mouse mixed brain cultures in a 96-well plate. The cultures were maintained in Dulbecco’s modified Eagle’s medium supplemented with 10% fetal bovine serum and antibiotics. Cytopathic effect (CPE) was assessed and the cultures were stained for reactivated viral expression using X-gal.

### 4.11. Nanostring Analysis 

Total RNA was extracted from the brains of the BALB/c animals latently infected with MCMV using a Qiagen RNeasy Lipid Tissue Extraction kit. The nCounter single molecule counting and digital quantification was then performed at the University of Minnesota Genomics Center. This technology is based on gene-specific probe pairs that are hybridized in a single multiplexed reaction to the sample in combination with automated imaging and detection; this eliminates enzymatic reactions that might bias the results. Data generated using the nCounter were then analyzed by our laboratory using the nSolver (version 4) and Rosalind online platform software tools. 

### 4.12. Statistical Analysis

One-way analysis of variance (ANOVA) with Tukey’s multiple comparison test was used for graphical analysis. Differences were considered significant when *p* < 0.05. For statistical analysis and generation of graphs, Prism 5 software (Version 10; GraphPad Software Inc., San Diego, CA, USA) was used.

## Data Availability

The original contributions presented in this study are included in the article. Further inquiries can be directed to the corresponding author(s).

## Abbreviations

The following abbreviations are used in this manuscript:

MCMV	Murine cytomegalovirus
bTRMs	Brain tissue-resident memory T-cells
LgalS3	Galectin 3
gpnmb	Glycoprotein nonmetastatic melanoma protein B
Hmox1	Heme oxygenase I
TREM2	Triggering receptor on myeloid cells 2
DAM	Disease-associated Microglia
MGnD	Neurodegenerative Microglia
NSC	Neural Stem cells
SVZ	Sub-ventricular zone
CTL	Cytotoxic T-cell
CNS	Central Nervous system
d.p.i	Days post infection
ICV	Intracerebroventricular
Ab	Antibody
Ag	Antigen
BMNCs	Brain mononuclear cells
HSV	Herpes Simplex virus
IFN	Interferon
FBS	Fetal bovine serum
TCID_50_	50% Tissue culture infective doses
CPE	Cytopathic effect
HPRT	Hypoxanthine phosphoribosyl transferase
PBS	Phosphate-buffered saline
RT	Room temperature
